# Application of *Propionibacterium* and *Lactobacillus* Starter Cultures in Semidry Fermented and Smoked Sausage Production: Effects on Quality, Safety, and Nitrite Reduction

**DOI:** 10.1155/ijfo/4733962

**Published:** 2025-09-23

**Authors:** Sholpan Baytukenova, Ulzhan Ryspaeva, Saule Baytukenova, Anel Kostanova, Saule Yeraliyeva

**Affiliations:** ^1^ Department of Food Technology and Processing Products, S. Seifullin Kazakh Agrotechnical Research University, Astana, Kazakhstan; ^2^ Department of Technology and Standardization, K. Kulazhanov Kazakh University of Technology and Business, Astana, Kazakhstan; ^3^ Department of Design and Technology, Korkyt Ata Kyzylorda University, Kyzylorda, Kazakhstan, korkyt.edu.kz

**Keywords:** amino acid composition, nitrite reduction, *Propionibacterium shermanii*, semidry fermented sausage, starter cultures

## Abstract

This study explores the functional role of *Propionibacterium shermanii* and *Lactobacillus acidophilus* as starter cultures in the development of semidry fermented and smoked sausages, with a focus on improving product quality and microbiological safety and reducing sodium nitrite levels. Four sausage formulations were prepared: one control without bacterial inoculation and three experimental groups containing different concentrations (0.08%, 0.10%, and 0.15%) of the bacterial blend. Results demonstrated that a 0.10% inoculation level achieved optimal fermentation, lowering the pH to 5.3 within 8 h and facilitating a 40% reduction in sodium nitrite without compromising color stability or safety. The inoculated samples exhibited significant improvements in water‐binding and holding capacities, as well as enhanced moisture regulation and accelerated drying. Microbiological analysis revealed effective suppression of spoilage organisms and absence of *E. coli*, while organoleptic evaluation confirmed superior texture, aroma, and visual appeal in the treated groups. Colorimetric assessment showed increased pinkness and brightness values, especially in the 0.10% group. Amino acid profiling indicated a 24.2% increase in essential amino acid content, including lysine, leucine, and threonine, due to enhanced proteolysis and microbial activity. The added cultures also contributed to a significant reduction in residual nitrite levels and supported nitrosopigment formation. Storage studies confirmed the product’s microbiological and physicochemical stability over 20 days under refrigeration. These findings highlight the potential of selected starter cultures as clean‐label alternatives to chemical preservatives, offering a promising approach for safer, nutritionally enriched, and high‐quality fermented meat products.

## 1. Introduction

Fermented meat products are highly valued for their distinctive flavor, improved preservation, and consumer appeal. These desirable attributes result from biochemical and microbiological processes that occur during fermentation, which enhance product safety, stability, and taste. The inclusion of starter cultures, especially lactic acid bacteria (LAB), plays a central role in this transformation by accelerating acidification, suppressing harmful microbes, and supporting the development of flavor and aroma.

In addition to LAB, propionic acid bacteria (PAB) have attracted interest as functional agents in food fermentation. While they are primarily used in the dairy sector—where they contribute to flavor development, gas formation, and the production of propionic and acetic acids—recent studies suggest their potential in meat processing as well. For example, *Propionibacterium freudenreichii* is recognized for generating flavor‐active branched‐chain compounds and contributing to the ripening of Swiss‐type cheeses [1–3]. Although underexplored in meat systems, PAB have demonstrated a range of beneficial effects, including antimicrobial activity, vitamin B12 synthesis, and pH reduction [4–6]. Their unique metabolic profile makes them promising candidates for enhancing both safety and nutritional value in fermented meat products.

A major concern in processed meats is the use of sodium nitrite (NaNO₂), which is essential for microbial control and color development but also associated with the formation of N‐nitrosamines, compounds considered potentially carcinogenic [7]. This has prompted a growing demand for natural alternatives that reduce chemical input without compromising quality. Recent research has examined the use of microorganisms to perform key functions traditionally achieved by nitrites. For instance, yeasts such as *Debaryomyces hansenii* have been used to maintain flavor at reduced nitrite levels [8], while some coagulase‐negative staphylococci (CNS) have shown promise as nitrite replacers [9]. LAB and PAB are also being studied for their ability to support product quality while mitigating health risks through nitrite degradation and suppression of harmful compounds [10, 11].

Starter cultures influence multiple parameters during sausage production, including acidification, microbial balance, and enzymatic breakdown of proteins and fats. These activities lead to the release of peptides, amino acids, and volatile compounds responsible for the characteristic flavor and improve the nutritional profile [4]. Moreover, their antimicrobial effects contribute to a longer shelf life and improved safety through competitive exclusion and the production of bacteriocins [11, 12].

Although LAB are routinely used in sausage fermentation, *Propionibacterium* spp. remain largely untapped as functional bacterial strains in this application, especially for health‐driven reformulation. To address this research gap, the present study evaluates how different concentrations (0.08%, 0.10%, and 0.15%) of *Propionibacterium shermanii* and *Lactobacillus acidophilus* affect the physicochemical, microbiological, and sensory characteristics of semidry fermented and smoked sausages. Parameters such as pH change, water activity, residual nitrite, nitrosopigment formation, amino acid enhancement, and organoleptic quality are assessed. The findings are aimed at supporting the use of these cultures as clean‐label, functional alternatives to conventional preservatives in meat products.

In this study, the focus is on a semidry fermented and smoked sausage, a product type that undergoes brief fermentation, followed by thermal processing and smoking. This processing model inactivates microbial cells while preserving their biochemical effects, making the product suitable for refrigerated storage rather than shelf stability. The aim is to examine how *P. shermanii* and *L. acidophilus* influence quality, microbial safety, and nitrite reduction when used as starter cultures in this formulation.

## 2. Materials and Methods

### 2.1. Sample Collection

First and second category beef, raw fat, and egg powder were selected as primary raw materials for this study. The first‐category beef, characterized by its higher lean content, and second category beef, known for contributing to desired texture, were supplied from certified farms in Astana, Kazakhstan, and stored at 4°C until further use. Raw fat was used to enhance the juiciness and mouthfeel of the sausages, while egg powder functioned as a binding agent to improve product consistency. Spices, including common salt (NaCl), sugar, and black pepper, were obtained from local markets in Astana and stored under dry conditions. NaNO_2_ was incorporated as a preservative in varying concentrations to study its reduction in the presence of starter cultures. A strain of PAB (*P. shermanii*) and LAB (*L. acidophilus*) was obtained in lyophilized form (Vector‐BiAlgam, Kol’tsovo, Russia). All chemicals used in the study were of analytical grade, ensuring the integrity and reproducibility of the experimental results.

### 2.2. Starter Culture Preparation

Both *P. shermanii* and *L. acidophilus* strains were reactivated prior to inoculation. Each strain was cultured separately in MRS broth under anaerobic conditions at 37°C for 24 h. The active cultures were then centrifuged, washed, and resuspended in sterile saline solution to achieve a final inoculum concentration of 10^8^ CFU/mL. A 1:1 ratio of *P. shermanii* and *L. acidophilus* was used to prepare the mixed inoculum, which was added to the sausage formulations at three concentrations: 0.08%, 0.10%, and 0.15%. This preparation protocol aligns with standard practices described by [5] for preparing starter cultures in fermented meat systems.

### 2.3. Sausage Formulation

Four formulations of semidry fermented and smoked sausage were developed for this study: one control (100% first‐category beef) and three experimental samples containing varying proportions of first‐ and second category beef with different concentrations of starter cultures (Table 1). This product type is characterized by a short fermentation phase, followed by thermal treatment and smoking, and requires refrigerated storage, distinct from dry fermented or fully cooked sausages. All processing was conducted following industry‐standard guidelines at the Department of Technology and Standardization, K. Kulazhanov Kazakh University of Technology and Business, Astana, Kazakhstan. The meat (Grades I and II) was ground using a meat grinder (TC22 Elegant Plus, Sirman, Italy) equipped with a 16–25 mm perforated plate. NaNO_2_ concentrate was added during this stage. The ground mixture was then combined with raw fat, egg powder, and spices and then homogenized in a vacuum mixer (Mainca RM‐20, Mainca, Barcelona, Spain) to ensure uniform distribution of ingredients. The temperature of the minced meat was maintained between −1°C and 5°C during processing, and the cutter loading factor was set between 0.3 and 0.6. Starter cultures (*P. shermanii* and *L. acidophilus*) were added postgrinding and prior to salting to allow uniform dispersion. Fermentation was carried out at 18°C for 8 h in a controlled fermentation chamber (KOMA KTI‐Q, KOMA Koeltechnische Industrie, Netherlands). To avoid microbial inhibition, no sodium chloride (NaCl) was added during fermentation. Following fermentation, salting was performed by immersing the matured meat in a salt solution at 10°C–12°C for 3 h, using 15%–20% of the meat raw material mass in a refrigerated water bath (Julabo F12‐ED, JULABO GmbH, Seelbach, Germany). Subsequently, the salted meat mixture was processed in a bowl cutter (Seydelmann K20, Maschinenfabrik Seydelmann KG, Stuttgart, Germany) for 5–10 min, ensuring the temperature did not exceed 12°C. The final sausage batter was then filled into fibrous casings (Ø 45 mm, 600 g of product per unit; Amiflex T, JSC “Letpaka,” Šiauliai, Lithuania) using a vacuum filler (HP‐25, Vemag Maschinenbau, Verden, Germany). The formed loaves were precured for 3 h at 4°C–8°C in a refrigerated chamber (Liebherr LGUex 1500, Liebherr Group, Bulle, Switzerland). The sausages then underwent thermal treatment, including roasting at 80°C–85°C for 30–40 min until the internal temperature reached 45°C, followed by steaming in steam chambers or boilers at 80°C–85°C for 40–60 min until the internal temperature reached 71^°^C ± 1^°^C, which was monitored using a digital meat thermometer (Testo 105, Testo SE & Co. KGaA, Lenzkirch, Germany). After thermal treatment, the sausages were chilled to 20°C for 30–40 min. Smoking was conducted at 45°C–50°C for 60–90 min in a smoking chamber (Fessmann T1900, Fessmann GmbH, Winnenden, Germany). The sausages were then dried at 10°C–12°C with air exchange for 36 h under 75%–85% humidity using a climate‐controlled drying unit (Reich AIRMASTER UKQ AIRJET, Reich Klima‐Räuchertechnik GmbH, Germany). Upon passing quality control, the sausages were packaged, labeled, and stored at approximately 5°C in a controlled temperature refrigeration unit (Liebherr LGUex 1500, Liebherr Group, Bulle, Switzerland) for up to 5 days prior to analytical testing, including pH, color, texture, amino acid composition, residual nitrite content, and microbiological stability. Each experimental formulation and the control were produced in three independent batches (*n* = 3) on separate days to ensure reproducibility.

**Table 1:** Composition of semidry fermented and smoked sausage samples.

**Ingredient**	**Control**	**Sample 1**	**Sample 2**	**Sample 3**
Beef Grade I (kg)	85	55	55	55
Beef Grade II (kg)	—	30	30	30
Raw fat (kg)	10	10	10	10
Egg powder (kg)	5	5	5	5
Total	100	100	100	100
*Spices (per 100 kg of raw materials)*				
Salt (kg)	1	1	1	1
Sugar (kg)	0.1	0.1	0.1	0.1
Black pepper (kg)	0.1	0.1	0.1	0.1
Sodium nitrite (g/100 kg)	1	0.8	0.6	0.4
*Starter microorganisms*				
*Propionibacterium shermanii*, *Lactobacillus acidophilus* (1:1)	—	0.08	0.1	0.15

### 2.4. Preparation of Minced Meat Samples for pH and Moisture Evaluation

To evaluate the acidification dynamics and moisture loss associated with *P. shermanii*, separate test samples of minced beef were prepared independently from the main sausage formulations. First‐category beef was minced using a grinder with a 16–25 mm diameter plate. Three experimental groups were created by inoculating the meat with propionic acid bacterium concentrate at concentrations of 0.08%, 0.10%, and 0.15%. To avoid inhibiting bacterial activity, neither sodium chloride (NaCl) nor NaNO_2_ was added. The mixtures were homogenized, vacuum‐sealed in sterile plastic bags, and incubated at 18°C for 24 h. Samples were collected at specific intervals—0, 4, 6, 8, 12, 14, 16, and 24 h—for the measurement of pH and moisture content. These evaluations enabled the determination of the most effective bacterial concentration for achieving optimal acidification while monitoring associated changes in water loss.

### 2.5. Proximate Composition

The chemical composition of sausage samples was assessed using a series of standardized analytical methods according to State Standard (ST GOST) methods. Moisture content was quantified by oven‐drying at 105°C to constant weight (GOST R 51479‐99, [13]), utilizing an HZ‐2014B oven (China). Protein content was measured using a Kjeldahl apparatus (UDK‐149, VELP Scientifica Srl, Italy), following GOST 25011‐81 [14], suitable for precise food protein determination. Ash content was established with the aid of a muffle furnace (LSM01, SNOL 8,2/1100, Lithuania), which operates at temperatures up to 1100°C, adhering to GOST 31727‐2012 [15]. Fat content analysis was carried out using a semiautomatic fat extractor SER 148/6 (VELP Scientifica Srl, Italy) in line with GOST 23042‐86 specifications [16]. The fatty acid profile of the samples was examined via gas chromatography, employing the Intuvo 9000 Gas Chromatography System (United States) according to methodologies outlined in GOST R 55483‐2013 [17] and cross‐referenced with GOST R 34132‐2017 [18]. Kazakhstan’s official standards are based on GOST regulatory guidelines, which are widely applied across Central Asia and CIS countries. These standards are publicly accessible via official platforms, including https://www.gostinfo.ru. All analyses were performed in triplicate to ensure the accuracy and consistency of the results.

### 2.6. Water‐Binding Capacity (WBC), Water‐Holding Capacity (WHC), pH Measurement, and Water Activity (*a*_*w*_)

The WBC of the samples was determined by the pressing method (filter paper) [19]. In brief, test samples weighing 0.30 ± 0.01 g were weighed on laboratory scales on a polyethylene circle with a diameter of 15–20 mm. The sample was then transferred to an ash‐free filter with a diameter of 9–11 cm, placed on a glass or plexiglass plate, ensuring that the sample was positioned under the polyethylene circle. The sample was covered with another plate of the same size, and a 1 kg weight was placed on top for 10 min. After this period, the filter with the sample was removed from under the weight and the upper plate. A pencil was used to outline the contour of the spot formed around the pressed sample. The contour of the wet spot appeared as the filter paper dried in the air. The area of the spot formed by adsorbed moisture was calculated by the difference between the total spot area and the area of the spot formed by the test sample. The areas of spots formed by the pressed sample and adsorbed moisture were measured using a planimeter. One square centimeter of the wet spot area on the filter corresponds to 8.4 mg of water. The mass fraction of bound moisture in the sample, expressed as a percentage of the sample mass, was determined using the following formula:

B=A−K∗B∗100M,

where *A* is the total moisture mass in the sample (milligram), *K* = 8.4 is the water content in 1 cm^2^ of the wet spot (milligram), *B* is the wet spot area formed by adsorbed moisture (square centimeter), and *M* is the sample weight of the meat (milligram).

The moisture released was quantified using digital imaging software “Compass‐3D V‐10.”

WHC was assessed using a standard gravimetric centrifuge method as described by Kristensen and Purslow [20]. The WHC was calculated using the following equation:

WHC %=M2−MM1−M×100,

where *M* represents the initial mass of the meat sample (gram), *M*_1_ is the mass of the meat sample after heating and removal of released moisture (gram), and *M*₂ is the mass of the minced meat sample after centrifugation and removal of separated moisture (gram).

The pH of the samples was determined using a potentiometric method with a pH meter (Model 340, Mettler‐Toledo GmbH, Switzerland) according to GOST R 51478‐99 [21]. To measure the pH, 5 g of the sample was blended with 20 mL of distilled water in a homogenizer (X–1000, United States) at 8000 rpm for 60 s. The mixture was allowed to equilibrate at 20°C for 30 min before recording the pH values.

Water activity was assessed in triplicate using a HygroLab C1 water activity meter (Rotronic, Bassersdorf, Switzerland). The measurements were conducted in AWQ mode, with samples allowed to stabilize for 20 min after reaching room temperature.

### 2.7. Determination of Amino Acid Profile and Fatty Acid Composition

The amino acid composition of the product was analyzed using ion exchange chromatography on a Biochrom 30+ automated analyzer following acid hydrolysis. The sample hydrolysis was carried out in a 6 N hydrochloric acid solution, ensuring complete breakdown of proteins into individual amino acids for precise quantification. The fatty acid composition was assessed by first extracting lipids from the paste samples using the chloroform/methanol extraction method, following the Folch procedure. The purity of the isolated lipids was confirmed via thin‐layer chromatography (TLC). Subsequently, the fatty acid profile was determined using a Hewlett Packard HP 6890 gas chromatograph equipped with a flame ionization detector (FID). The analysis was conducted on an HP‐Innowax capillary column (30 m × 0.25 mm × 0.25 *μ*m), manufactured by Agilent Technologies, United States, ensuring high‐resolution separation and identification of fatty acids in the sample.

### 2.8. Determination of Microbiological Parameters, Residual Nitrite, Nitrosopigment Content, and Organoleptic Evaluation

Microbiological assessment of the product was carried out by bacteriological analysis according to GOST 51448‐2010 [22]. The following indicators were determined: the number of mesophilic aerobic and facultative anaerobic microorganisms (MAFAnMs), lactic acid microorganisms, and bacteria of the *Escherichia coli* (*E. coli*) group.

The quantification of residual nitrite levels was performed using the Griess reaction, which is based on the interaction of nitrous acid with *α*‐naphthylamine and sulfanilic acid in the presence of acetic acid. This reaction forms a red‐colored compound, the optical density of which is measured using a UV‐1800 Spectrophotometer (Shimadzu UV‐1800, 115 VAC, Shimadzu, Japan) at a wavelength of 540 ± 2 nm. The method allows for precise quantification of nitrite levels in various meat products, including sausages, semifinished products, and poultry. To calculate the nitrite levels, the following formula is used:

X=C∗20010030∗∗m∗205∗∗106,

where *C* is the concentration of NaNO_2_ determined from the calibration curve in micrograms per cubic centimeter, 200 is the volume to which the sample is diluted (cubic centimeter), 100 is the filtrate volume (cubic centimeter), 30 is the total volume used for the colorimetric reaction (cubic centimeter), *m* is the mass of the analyzed sample (gram), 20 is the filtrate volume used for protein precipitation (cubic centimeter), 5 is the volume of the filtrate used for the colorimetric reaction (cubic centimeter), and 10^6^ is the conversion factor from micrograms to grams.

The content of nitrosopigments and the total amount of pigments were determined by the method based on the extraction of pigments of meat and meat products with aqueous acetone solution, followed by measurement of the optical density of the extract on the photoelectrocolorimeter KF‐77 at 540 nm.

A sensory evaluation of the final sausage products was carried out by a panel of 15 untrained but regular consumers of sausages, using the methodology defined by GOST 1731‐2007 [23]. A structured 5‐point hedonic scale was employed to assess attributes such as appearance, color, aroma, texture, sectional view, and overall acceptability. On this scale, a rating of 5 corresponded to *extremely like* with ideal sensory properties, while a rating of 1 indicated *extremely dislike*. Each panelist received coded samples of the control product along with three test variants (Samples 1, 2, and 3), which contained 0.08%, 0.1%, and 0.15% starter culture blends, respectively. All samples were sliced uniformly, served at room temperature under consistent lighting, and presented in randomized order to minimize evaluator bias. To cleanse the palate between samples, participants were given plain water and unsalted bread [24]. Employing untrained panelists in early sensory assessment aligns with established protocols in meat product reformulation, where initial testing seeks to identify noticeable sensory differences before broader consumer validation [25].

### 2.9. Statistical Analysis

Each experimental formulation and the control were produced in three independent treatments (*n* = 3) on separate days to ensure reproducibility. Data were analyzed using one‐way ANOVA followed by Tukey’s HSD test for multiple comparisons (*p* < 0.05) using SPSS software (IBM SPSS Statistics 26). Results are presented as mean ± standard deviation (SD).

## 3. Result and Discussion

### 3.1. Selection of Cultivation Conditions for Propionic Acid Microorganisms in Minced Meat

Meat provides a nutrient‐rich environment conducive to microbial proliferation. In the context of fermented sausage production, the selection of bacterial strains depends significantly on their ability to survive and function under stress conditions imposed by preservation agents like NaNO_2_ and sodium chloride. Previous studies have demonstrated that specific strains of *Lactobacillus* possess efficient nitrite‐reducing activity, while others show notable salt tolerance, enabling them to thrive during meat fermentation processes [26, 27]. Before incorporating the starter cultures into the sausage formulation, preliminary in vitro experiments were conducted to assess the resistance of *P. shermanii* to different concentrations of sodium chloride and NaNO_2_. These trials are aimed at ensuring that the selected strains could remain metabolically active under conditions commonly encountered in fermented meat products, even though such concentrations were not applied directly in the final product formulation. Sodium chloride and NaNO₂ are critical curing agents in sausage production, but they can inhibit microbial growth if applied before fermentation. To avoid such inhibition in the actual sausage preparation, the starter cultures were added prior to sodium chloride addition to allow unhindered microbial fermentation. The salting step was performed only after the 8‐h fermentation period. Table 2 presents the results of the sodium chloride tolerance experiment. The bacteria demonstrated robust growth across all tested concentrations. At 2.5% sodium chloride, the microbial count reached 4 × 10^10^ CFU/g, and further increases were observed at 3.0% and 3.5%. Statistical analysis showed a significant effect of sodium chloride concentration on bacterial proliferation (*p* < 0.01), with Tukey’s post hoc analysis confirming that bacterial growth at 3.0% and 3.5% was significantly higher than at 2.0% and 2.5% (*p* < 0.05). These findings support previous research on salt tolerance in lactic and PAB in fermented meat products [27, 28].

**Table 2:** Growth of *Propionibacterium shermanii* at different sodium chloride concentrations.

**Sodium chloride concentration (%)**	**Growth (CFU/g)**
2.0	2 × 10^10^
2.5	4 × 10^10^
3.0	6 × 10^10^
3.5	1 × 10^11^

NaNO₂ plays a crucial role in enhancing the sensory properties of the final product, acting as an antioxidant and inhibiting the growth of harmful microorganisms, which ultimately extends shelf life [7, 10]. NaNO₂ resistance was evaluated separately, with results summarized in Table 3. Statistical analysis revealed a significant effect of NaNO₂ concentration on bacterial growth (*p* < 0.05). At 4 mg/100 g, the bacterial count was highest (33 × 10^10^ CFU/g), whereas at 8 mg/100 g, a marked inhibition was observed (7 × 10^10^ CFU/g). Optical density readings corresponded with these trends, showing a peak at 6 mg/100 g (1.83 ± 0.046). Statistical analysis confirmed a significant effect of nitrite level on bacterial growth (*p* < 0.05). Similar inhibitory effects of NaNO₂ on microbial growth have been reported in other studies on fermented meat, while moderate nitrite concentrations have also been shown to suppress bacterial proliferation and promote desirable microbial activity, thereby effectively balancing safety and quality during sausage production [7, 29–31].

**Table 3:** Effect of sodium nitrite concentration on *Propionibacterium shermanii* growth.

**Sample**	**Sodium nitrite level (mg/100** g**)**	**Optical density (OD)**	**Growth of propionic acid microorganisms (CFU/g)**
Reference sample	—	1.70 ± 0.063	3 × 10^11^
Sample 1 (0.08%)	8	1.59 ± 0.030	7 × 10^10^
Sample 2 (0.10%)	6	1.83 ± 0.046	14 × 10^10^
Sample 3 (0.15%)	4	1.73 ± 0.022	33 × 10^10^

*Note:* Optical density (OD) was measured at 600 nm as an auxiliary indicator of microbial proliferation. CFU/g values were used as the primary measure of bacterial growth, while OD trends supported the inverse relationship observed between nitrite concentration and bacterial viability.

These tolerance findings were used to inform strain selection and inoculation levels. Although NaNO₂ (1 g/100 kg) was included during grinding, sodium chloride, which exerts a stronger inhibitory effect on bacterial metabolism, was added only after fermentation. This approach ensured that the acidification activity of the starter cultures was not significantly compromised, allowing for effective pH reduction during the fermentation phase.

### 3.2. Biochemical Changes in Minced Meat With PAB

**Figure 1:**
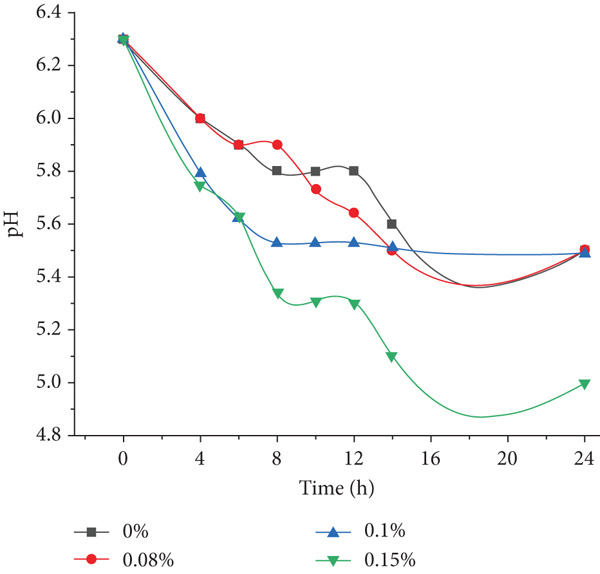
Dynamics of pH reduction in minced beef with different concentrations of *Propionibacterium shermanii* during 24‐h fermentation.

The initial stage of this study is aimed at determining the optimal concentration of *P. shermanii* and *L. acidophilus* for use as starter cultures in semidry fermented and smoked sausage production. Experimental minced beef samples were inoculated with three concentrations of the bacterial blend, 0.08%, 0.1%, and 0.15%, and monitored over 24 h under controlled conditions to evaluate their effects on pH and moisture content. Figure 1 illustrates the temporal pH evolution across all treatments. A rapid pH decline was observed during the initial 12–18 h of fermentation in all inoculated samples, consistent with the acidifying action of propionic and lactic acid–producing bacteria.

Among the groups, the 0.1% starter concentration exhibited the most desirable acidification pattern, reaching a pH of 5.3 within 8 h and stabilizing thereafter, which aligns with optimal values for semidry sausage fermentation. In contrast, the 0.15% group (Sample 3) showed the most pronounced pH drop, reaching 5.0 after 12 h, followed by a mild increase in pH by 24 h. Statistical analysis confirmed the significance of bacterial concentration on pH reduction (ANOVA, *p* < 0.05). Post hoc testing revealed that both the 0.1% and 0.15% groups produced significantly lower pH values than the control and 0.08% groups after 8 h (Tukey’s test, *p* < 0.05). These results align with previous studies on the pH‐lowering effects of PAB in fermented meat products [32–34].

After an initial sharp decline during the first 12–18 h, a modest pH rebound was observed (Figure 1), particularly in the 0.15% group. This effect may reflect proteolytic activity or accumulation of basic nitrogenous compounds, such as ammonia and amines, arising from early protein hydrolysis, which can occur in overacidified systems or during prolonged fermentation without further microbial control. Since our fermentation lasted only 8 h in the final sausage production process, this rebound does not occur in the actual product but was observed under extended experimental conditions for evaluation purposes. Although starter cultures were applied, the product cannot be classified as a traditional dry fermented sausage due to the short fermentation period (8 h) and subsequent heat treatment. The process corresponds to a semidry fermented sausage, in which the functional role of the cultures is confined to early acidification and color stabilization prior to microbial inactivation through cooking.

**Figure 2:**
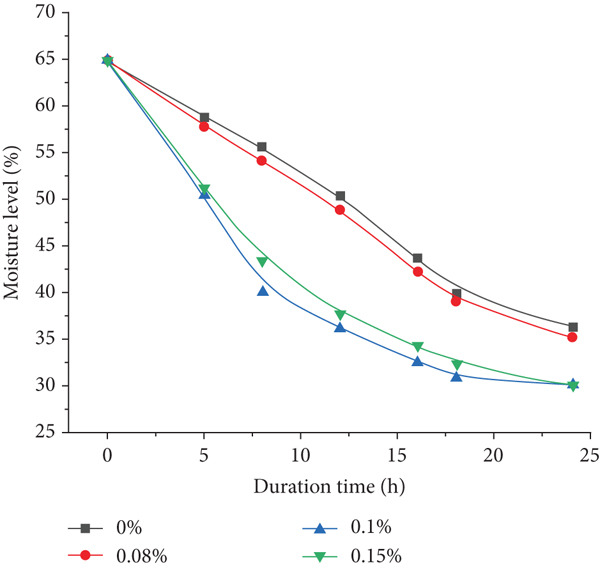
Effect of *Propionibacterium shermanii* concentration on moisture loss in minced meat during 24‐h fermentation.

The moisture content was measured to assess the interaction between acidification and water loss. As shown in Figure 2, all samples experienced a steady reduction in moisture over the 24‐h period, with the most substantial loss observed in Sample 3 (0.15%), reaching approximately 32%. This moisture reduction supports improved texture, as bacterial acidification promotes protein denaturation and water expulsion. Moisture retention was highest in the control group, with intermediate values for Samples 1 and 2. Statistical analysis revealed a significant effect of bacterial concentration on moisture reduction (*p* < 0.01), supporting the conclusion that acidification enhances dehydration efficiency. Similar results were observed in studies on moisture dynamics in fermented sausages during starter‐driven fermentation, where microbial acidification and temperature accelerated water loss, measurably reducing moisture through the drying phase, thereby enhancing textural firmness and product stability [35, 36].

Taken together, these results indicate that the 0.1% concentration of *P. shermanii* and *L. acidophilus* offers an optimal balance of pH reduction and moisture control without inducing excessive acidification or proteolytic instability. Consequently, this concentration was selected for the main sausage formulation in subsequent phases of the study.

Based on the data obtained, it can be concluded that the treatment of raw materials with PAB (*P. shermanii* and *L. acidophilus*) has a significant positive impact on the ripening process of semidry fermented and smoked sausages. The observed changes in pH and moisture content are directly associated with microbiological activity, including the production of lactic acid and the gradual reduction in the WBC of muscle proteins. These biochemical transformations contribute to the development of a final product with improved texture, enhanced flavor, and increased resistance to microbial spoilage. The reduction in pH inhibits the growth of undesirable microorganisms, while the controlled moisture loss ensures better shelf stability and overall product safety, highlighting the effectiveness of PAB in fermented meat processing. Among the tested groups, a 0.1% concentration of PAB provided the best balance between acidity control and moisture reduction, supporting improved texture and microbiological safety.

To study the potential of PAB to enhance microbial stability in semidry fermented and smoked sausages, we analyzed changes in water activity (*a*_*w*_) during the incubation of inoculated minced beef (Table 4). Water activity is a crucial factor influencing microbial growth, and its reduction is closely associated with improved product safety and extended shelf life [36]. Among the tested groups, the sample with 0.15% *P. shermanii* and *L. acidophilus* exhibited the lowest *a*_*w*_ value (0.8210), compared to the control (0.9241), showing a statistically significant difference (*p* < 0.05). This group also recorded the most pronounced pH decline (to 5.0, Figure 1), suggesting strong microbial metabolic activity and enhanced water‐binding effects. Despite these advantages in microbial suppression, such intense acidification may not always be optimal for maintaining desirable texture and sensory properties. By contrast, the 0.10% inoculation group achieved a more moderate reduction in *a*_*w*_ and pH (~4.79), representing a balanced environment that favors microbial stability without excessive moisture loss. This concentration was therefore considered the most suitable for application in sausage production, offering improved safety while supporting favorable organoleptic outcomes. These observations align with earlier findings emphasizing the importance of optimizing both acidification and moisture dynamics to produce safe, high‐quality fermented meat products [36, 37].

**Table 4:** Water activity (*a*_*w*_) in minced beef with different starter culture concentrations during incubation.

**Duration (h)**	**Control**	**Sample 1 (0.08%)**	**Sample 2 (0.1%)**	**Sample 3 (0.15%)**
0	0.9608 ± 0.0032	0.9438 ± 0.0028	0.9441 ± 0.0031	0.9421 ± 0.0030
5	0.9578 ± 0.0034	0.9382 ± 0.0030	0.9353 ± 0.0027	0.9307 ± 0.0025
8	0.9501 ± 0.0030	0.9305 ± 0.0026	0.9152 ± 0.0024	0.9112 ± 0.0022
12	0.9482 ± 0.0033	0.9230 ± 0.0028	0.9034 ± 0.0029	0.9021 ± 0.0027
18	0.9301 ± 0.0029	0.9101 ± 0.0025	0.8652 ± 0.0021	0.8642 ± 0.0020
24	0.9241 ± 0.0028	0.9049 ± 0.0024	0.8222 ± 0.0020	0.8210 ± 0.0019

*Note:* Data are expressed as mean ± standard deviation (SD) from triplicate measurements. Significant differences among samples were determined using ANOVA followed by Tukey’s HSD test (*p* < 0.05).

The effectiveness of NaNO₂ in developing and stabilizing color in meat products was evaluated by measuring nitrosopigment formation and color stability (Table 5). PAB exhibit nitrite reductase, facilitating the conversion of NaNO₂ to nitric oxide (NO), which binds to myoglobin and promotes stable nitrosopigment formation responsible for the characteristic cured meat color. The experimental results demonstrated that the treatment of raw materials with PAB significantly increased nitrosopigment formation and improved color stability. Even with a 40% reduction in NaNO₂ (from 10 to 6 g/kg of raw material), the amount of nitrosopigments in the treated samples was 5% higher than in the control, confirming the role of bacterial metabolism in nitrosomyoglobin formation and improved color characteristics [34, 38]. Sample 2 (0.1% bacteria) exhibited the highest nitrosopigment percentage (80.5%) and optimal color stability (75%), indicating that this bacterial concentration balances nitrite reduction with visual appeal (*p* < 0.05). Meanwhile, Sample 3 (0.15%), despite having a greater moisture reduction, displayed lower nitrosopigment formation, suggesting that higher bacterial concentrations may not always be beneficial. The reduction in nitrite levels and the bacterial‐driven color stabilization further support the potential for safer and healthier fermented meat products. Previous studies indicate that during fermentation of dried sausage, up to 25% of nitrates are naturally reduced through carbohydrate interactions, further improving product safety and nutritional quality [32, 33, 39, 40].

**Table 5:** Nitrosopigment content and color stability.

**Sample**	**Nitrite (mg/100** g**)**	**Nitrosopigment (% of total pigment)**	**Color stability (%)**
Control (0%)	10 ± 0.5	73.6 ± 2.1	75 ± 2.3
Sample 1 (0.08%)	8 ± 0.4	75.8 ± 2.0	72 ± 2.1
Sample 2 (0.1%)	6 ± 0.3	80.5 ± 1.9	75 ± 1.8
Sample 3 (0.15%)	4 ± 0.2	72.89 ± 1.8	70 ± 1.9

*Note:* Data are expressed as mean ± standard deviation (SD) from triplicate measurements. Significant differences among samples were determined using ANOVA followed by Tukey’s HSD test (*p* < 0.05).

While moisture loss can influence pigment concentration through reduced water activity, the observed increase in nitrosopigment formation was more closely associated with the metabolic activity of *P. shermanii*. This species possesses constitutive nitrite reductase, enabling the conversion of added NaNO₂ into NO, which facilitates nitrosopigment synthesis. Notably, samples with comparable moisture content exhibited varying degrees of pigment intensity, suggesting that enzymatic nitrite reduction played a more prominent role than dehydration alone. Further studies correlating water loss, residual nitrite, and pigment formation are warranted to strengthen these findings.

**Figure 3:**
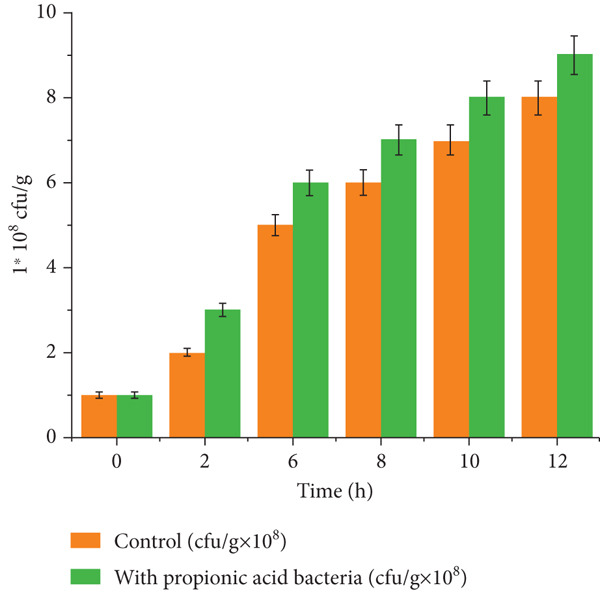
Quantification of propionic acid microorganisms in minced meat during salting at 12°C.

After the preliminary studies, the quantification of propionic acid microorganisms was conducted at a salting temperature of 12°C to monitor microbial growth over time. The results, shown in Figure 3, indicate that microorganisms developed well in the meat medium. Significant differences in microbial growth between the control and experimental samples were observed throughout the salting process (*p* < 0.05). The sample with added PAB reached 1 × 10^8^ CFU/g at 8 h, while the control sample achieved the same level only after 12 h, indicating faster microbial growth and higher biochemical activity in the treated sample. These results are aligned with previous studies that reported enhanced microbial adaptation and increased biochemical activity in fermented meat due to the addition of PAB [39, 41, 42].

The enhanced growth of PAB highlights their adaptability and metabolic activity in the minced meat environment, which plays a critical role in improving fermentation efficiency and biochemical transformations during salting. These findings are consistent with similar research showing that microbial activity during salting contributes to rapid pH reduction and water activity control, resulting in improved product stability and extended shelf life [43–45]. This confirms that the inclusion of PAB significantly enhances the salting process, resulting in a more active and efficient microbial population compared to the control.

### 3.3. Technological Properties of Sausages Treated With Propionic Acid Microorganisms

**Figure 4:**
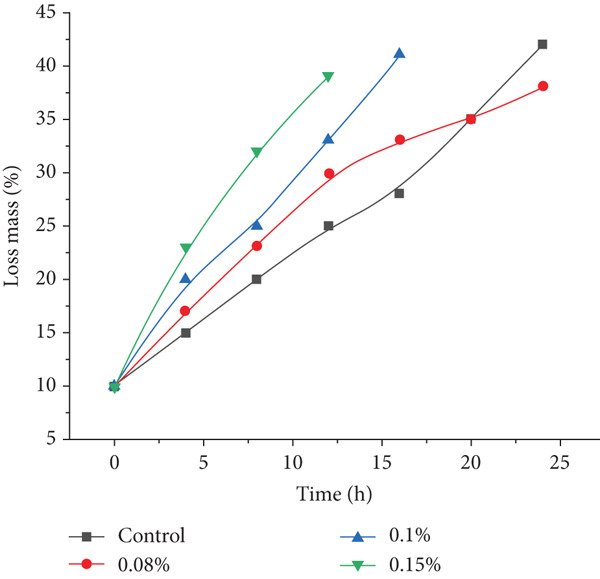
Mass loss dynamics during drying of semidry fermented and smoked sausages treated with propionic acid bacteria.

To evaluate the effects of *P. shermanii* and *L. acidophilus* on the physical properties of semidry fermented and smoked sausages, changes in mass loss during drying, WHC, and WBC were monitored. These parameters are essential for product yield, moisture regulation, and final texture quality. Figure 4 illustrates the mass loss trend over 24 h. All bacterial‐treated samples showed higher moisture loss than the control, with statistically significant differences (*p* < 0.05). Among the treated groups, Sample 3 (0.15% inoculum) exhibited the fastest dehydration, reaching a mass loss of 32% at 8 h, followed by Sample 2 (0.1%) at 25% and Sample 1 (0.08%) at 23%, compared to 20% in the control. The accelerated moisture evaporation in inoculated samples is attributed to reduced pH levels that shift protein structures towards their isoelectric point, reducing water retention capacity and enhancing fluid release [46, 47]. At the end of the drying period (24 h), Sample 3 displayed the highest cumulative mass loss (45%), while the control reached 42%. Although the initial drying rate was faster in inoculated samples, the final equilibrium moisture content was comparable, indicating that PAB mainly impact early drying stages.

In addition, WHC and WBC were assessed throughout drying (Table 6). These values declined progressively in all groups. However, the reduction was more pronounced in bacterial‐treated samples, indicating greater structural modification and moisture release. At 12 h, WHC in Sample 3 dropped to 74*%* ± 0.8*%*, significantly lower (*p* < 0.05) than the control (85*%* ± 0.8*%*). WBC followed a similar trend, with Sample 3 registering 72*%* ± 0.7*%*, compared to 80*%* ± 0.7*%* in the control group. By 24 h, WBC in Sample 3 declined to 61*%* ± 1.0*%*, confirming the influence of bacterial fermentation on enhancing water expulsion. These results suggest that incorporating PAB improves moisture regulation and promotes more efficient drying. The accelerated biochemical activity induced by microbial metabolism contributes to better structural adaptation, enhancing the physical stability and potential shelf life of fermented sausage products [32, 47, 48].

**Table 6:** Changes in water‐holding and water‐binding capacities during drying of semidry fermented and smoked sausages treated with propionic acid bacteria.

**Time (h)**	**WHC—control (0%)**	**WHC—Sample 1 (0.08%)**	**WHC—Sample 2 (0.1%)**	**WHC—Sample 3 (0.15%)**	**WBC—control (0%)**	**WBC—Sample 1 (0.08%)**	**WBC—Sample 2 (0.1%)**	**WBC—Sample 3 (0.15%)**
0	95 ± 0.5	94 ± 0.5	93 ± 0.5	92 ± 0.5	90 ± 0.4	89 ± 0.4	88 ± 0.4	87 ± 0.4
4	92 ± 0.6	90 ± 0.6	88 ± 0.6	86 ± 0.6	87 ± 0.5	85 ± 0.5	83 ± 0.5	81 ± 0.5
8	89 ± 0.7	86 ± 0.7	83 ± 0.7	80 ± 0.7	83 ± 0.6	81 ± 0.6	79 ± 0.6	76 ± 0.6
12	85 ± 0.8	81 ± 0.8	78 ± 0.8	74 ± 0.8	80 ± 0.7	77 ± 0.7	75 ± 0.7	72 ± 0.7
16	82 ± 0.9	77 ± 0.9	73 ± 0.9	69 ± 0.9	76 ± 0.8	73 ± 0.8	71 ± 0.8	67 ± 0.8
20	78 ± 1.0	74 ± 1.0	70 ± 1.0	66 ± 1.0	73 ± 0.9	70 ± 0.9	68 ± 0.9	64 ± 0.9
24	75 ± 1.1	71 ± 1.1	67 ± 1.1	63 ± 1.1	70 ± 1.0	68 ± 1.0	65 ± 1.0	61 ± 1.0

*Note:* Data are expressed as mean ± standard deviation (SD) from triplicate measurements. Significant differences among samples were determined using ANOVA followed by Tukey’s HSD test (*p* < 0.05).

### 3.4. Quality Characteristics of Sausages With PAB

**Figure 5:**
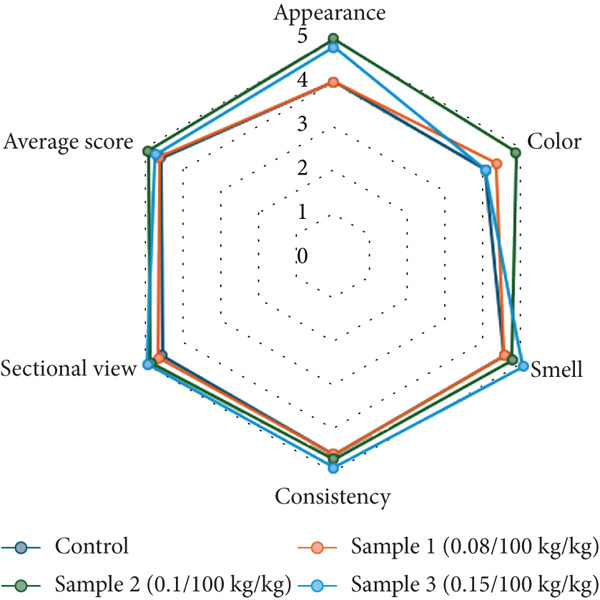
Organoleptic properties of semidry fermented and smoked sausages with propionic acid bacteria.

The final stage of the study involved evaluating the qualitative characteristics of semidry fermented and smoked sausages produced with PAB. The sensory evaluation of the sausage samples was performed in accordance with ST RK 1731‐2007 [23] using a structured 5‐point hedonic scale, where 5 represented *extremely like* and 1 indicated *extremely dislike*. Panelists assessed six distinct parameters to determine overall product acceptability. Appearance was evaluated based on visual appeal, surface uniformity, and casing integrity. Color referred to the intensity and uniformity of the red or pink shade associated with desirable curing. Smell involved the detection of a pleasant, typical aroma free of off‐odors. Consistency was judged by touch and mouthfeel, focusing on the firmness, cohesiveness, and chewiness of the sausage. The sectional view assessed the internal structure upon slicing, including fat distribution and binding. Finally, an average score was calculated to represent the combined sensory impression across all criteria. These parameters are illustrated in Figure 5, which visually compares the control and experimental groups to highlight the sensory impact of different starter culture concentrations.

The consistency of the experimental samples differed significantly (*p* < 0.05) from the control, exhibiting a denser and more monolithic structure. The color of Sample 2 (0.1% bacteria) was a uniform, intense red, indicative of enhanced nitrosopigment stability. In contrast, the control sample had a looser texture and a less vibrant color, suggesting lower protein gel formation and weaker curing effects [49]. Sample 3 (0.15% bacteria) developed a more acidic taste, sharper aroma, and darker red color, likely due to the higher concentration of LAB, which are known to contribute to flavor development in fermented meat products [50]. Figure 5 also demonstrates that Sample 3 (0.15%) achieved the highest overall organoleptic scores, followed by Sample 2 (0.1%), confirming the positive influence of PAB on sensory attributes. The control sample had the lowest ratings, reflecting weaker structural integrity, color intensity, and aroma development. The distinct aroma and taste profile in Sample 2 suggest that PAB enhance secondary metabolite production, which plays a crucial role in flavor development [34]. The organoleptic evaluation results align with the structural and mechanical properties, supporting the hypothesis that microbial composition influences texture, color, and sensory perception. These findings confirm that PAB contribute positively to the overall acceptability and technological quality of fermented meat products while also allowing for optimized moisture retention and structural stability.

In semidry fermented and smoked sausages, moderate moisture loss during fermentation and early drying is essential for achieving a firmer, cohesive texture. Unlike fresh or fully cooked sausages, where juiciness is often associated with better palatability, semidry products are traditionally appreciated for their sliceability and compact structure. This drier texture enhances mouthfeel and contributes to improved sensory scores for consistency and bite. Our results showed that the sample with 0.1% starter culture, which had a greater moisture reduction, was also rated highest for texture by the tasting panel. This suggests that controlled water loss supports optimal texture without causing undesirable dryness, striking a balance between firmness and residual juiciness preferred in this sausage category.

### 3.5. Color Attributes of Semidry Fermented and Smoked Sausages Enriched With PAB

**Figure 6:**
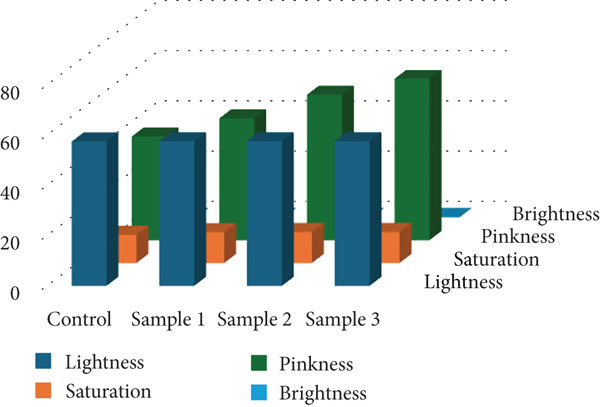
Color parameters (lightness, saturation, pinkness, and brightness) of semidry fermented and smoked sausages treated with propionic acid bacteria.

Color plays a pivotal role in consumer appeal and purchasing decisions for meat products. As shown in Figure 6, the lightness, saturation, pinkness, and brightness values were measured across all experimental groups, including control and samples inoculated with PAB. Statistical analysis revealed significant differences (*p* < 0.05) between control and treated samples, indicating that microbial fermentation enhances color development and pigment stabilization. Specifically, Sample 2 (0.1%) exhibited the most pronounced pink hue (63.9) and the highest brightness score (0.18), both significantly greater than those in the control group (*p* < 0.05), which showed a pinkness of 41 and brightness of 0.125. This enhancement is likely due to microbial activity influencing nitrosomyoglobin formation and pigment binding.

Although direct studies on *Propionibacterium* spp. and residual nitrite reduction are scarce, related research suggests that certain bacterial strains impact nitrite dynamics and meat coloration. For instance, Zhu and Yang [51] demonstrated the ability of LAB to express nitrite reductase, converting nitrite into NO. This compound binds with myoglobin, yielding a stable red pigment and contributing to reduced residual nitrite content. Similarly, other authors [32] have demonstrated that CNS facilitate nitrate‐to‐nitrite conversion followed by NO release, a key mechanism in stable cured color formation. These microbial transformations not only promote a desirable reddish color but also reduce reliance on added nitrites, supporting improved product safety and appeal.

Based on current findings, using 0.1% propionic acid bacterium concentrate contributes meaningfully to enhanced visual properties, reinforcing the functional role of starter cultures in both color development and nitrite management in semidry fermented and smoked sausages [32, 51, 52].

### 3.6. Residual Nitrite Reduction and Nitrosopigment Formation in Sausages

**Figure 7:**
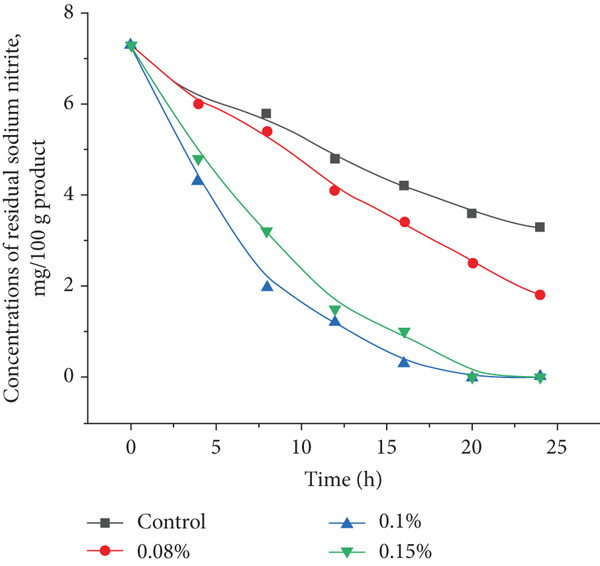
Reduction dynamics of residual nitrite in semidry fermented and smoked sausages treated with propionic acid bacteria.

The influence of PAB on residual nitrite content and nitrosopigment formation was evaluated to understand their contribution to the curing process in semidry fermented and smoked sausages. As shown in Figure 7, a significant decline in residual nitrite content was observed in all treated samples compared to the control (*p* < 0.05). The control retained 3.3 mg/100 g of residual nitrite after 24 h, remaining within the limit established by CU TR 034/2011 “On safety of meat and meat products” (5 mg/kg for meat products). However, samples inoculated with *P. shermanii* and *L. acidophilus* exhibited enhanced nitrite degradation. Sample 2 (0.1%) showed the fastest reduction, with nitrite levels dropping to 1.2 mg/100 g at 12 h and becoming undetectable by 20 h. Sample 3 (0.15%) reached complete nitrite depletion by 24 h. These results highlight the strong metabolic activity of the bacterial cultures in reducing nitrite to NO, which then binds to myoglobin and forms stable nitrosopigments—key compounds for achieving desirable cured color. Previous studies support this mechanism. Thus, Xu and Zhu [53] found that *Lactobacillus fermentum* and *Lactobacillus plantarum* effectively reduced nitrite content while preserving meat color in pork sausages. Similarly, Laranjo et al. [32] highlighted that CNS aid in nitrate‐to‐nitrite conversion and enhance color stabilization. These microbial transformations are essential for achieving uniform nitrosopigment formation in fermented meats. In addition to improving color, nitrites serve as antioxidants that help inhibit lipid oxidation, thereby extending product shelf life [54]. Moreover, research shows that up to 25% of nitrates in dry fermented sausages are converted by microbial pathways during carbohydrate metabolism [55]. These processes, accelerated by PAB, not only enhance the curing efficiency and pigment formation but also reduce nitrite residues, supporting safer and more natural meat processing practices [43, 56].

### 3.7. pH Dynamics in Semidry Fermented and Smoked Sausages During Storage

The pH level is a critical factor influencing the shelf life, microbial stability, and overall quality of semidry fermented and smoked sausages. Although the fermentation period in this study was limited to 8 h at 18°C, shorter than traditional protocols for dry fermented products, the use of *P. shermanii* and *L. acidophilus* effectively lowered the pH to optimal levels prior to thermal treatment. Subsequent roasting, steaming, and smoking raised the internal temperature to 71^°^C ± 1^°^C, ensuring inactivation of vegetative cells, including the inoculated bacteria. As a result, further microbial fermentation was not expected during storage.

**Figure 8:**
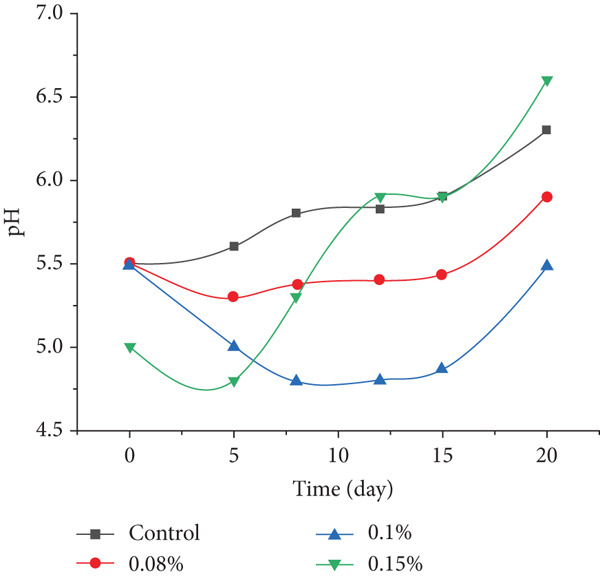
pH dynamics of semidry fermented and smoked sausages during 20‐day refrigerated storage: effects of different starter culture concentrations.

Nonetheless, as shown in Figure 8, statistically significant (*p* < 0.05) pH changes were observed throughout the 20‐day refrigeration period. These shifts are most likely due to postthermal biochemical processes such as protein degradation, residual enzymatic activity, and moisture redistribution, rather than microbial regrowth. Similar patterns have been reported in other thermally processed sausages, where modest pH fluctuations during cold storage were attributed to nonmicrobial mechanisms [57]. For instance, Stangierski et al. [45] documented a pH rise from 5.16 to 5.42 in smoked salami stored at refrigeration temperatures, reflecting ongoing biochemical transformations rather than spoilage. The average pH values observed in the present study align with those reported for Mediterranean‐style fermented sausages and support the conclusion that thermal and microbial treatments establish a stable biochemical environment during storage [58, 59].

### 3.8. Microbiological Stability of Semidry Fermented and Smoked Sausages During Refrigerated Storage

Microbiological safety is a critical factor in determining the quality and shelf stability of fermented meat products. To evaluate the microbiological stability of the semidry fermented and smoked sausages developed in this study, microbial counts were assessed during refrigerated storage over a 20‐day period (Table 7). Significant differences (*p* < 0.05) were observed between control and experimental groups, particularly in MAFAnM and LAB levels. Although the added starter cultures (*P. shermanii* and *L. acidophilus*) were likely inactivated during thermal processing (steaming at 80°C–85°C), their prefermentation metabolic activity, including the production of lactic acid and antimicrobial peptides such as bacteriocins, played a key role in suppressing spoilage organisms during the early storage phase. This was evidenced by the absence of *E. coli* in all experimental samples throughout the 20‐day storage, in line with food safety requirements outlined in Technical Regulation 021/2011 “On Food Safety”. By Day 7, elevated LAB counts were observed in all treated samples, indicating the persistence of native and surviving LAB populations that contributed to a stable microbial ecosystem. Peak microbial activity was recorded on Day 15, consistent with previous research indicating midstorage microbiota adaptation in fermented meats [32, 34]. Notably, Sample 2 (0.1% bacterial inoculum) maintained a more balanced microbial profile, showing a moderate decline by Day 20, suggesting better control of microbial succession and spoilage inhibition. This microbial behavior is consistent with previous findings, demonstrating that early‐stage acidification and metabolite formation can suppress pathogenic microflora and improve microbiological safety [32, 34]. For instance, *L. plantarum* has been shown to inhibit undesirable microbes via acidification and bacteriocin production during the storage of raw smoked sausages [32]. It is important to note that the primary purpose of this product is short‐term refrigerated storage, as described in the formulation section. The fermentation and thermal processing were optimized not for prolonged ambient shelf life, but for enhancing quality and safety during cold storage conditions (approximately 5°C for up to 5 days, with additional monitoring up to 20 days for assessment). Therefore, the microbiological stability observed is attributable more to residual antimicrobial metabolites and environmental LAB rather than to continuous activity of the added starter cultures, which are likely rendered nonviable after heat treatment. These findings confirm that early microbial intervention via starter culture fermentation contributes significantly to the safety and microbial balance of the product during its intended storage window, ensuring both regulatory compliance and extended freshness under refrigeration.

**Table 7:** Changes in microbiological parameters of model samples of semidry fermented and sausage during refrigerated storage.

**Samples**	**Time (24** h**), temperature storage** **m** **o** **d** **e** + 2^°^ **C** ± 4^°^ **C**	**Microbiological indicators**		
		**Number of mesophilic aerobic facultative anaerobic microorganisms (CFU/g)**	**Lactic acid microorganisms**	***E. coli* bacteria**
Control	0	0.1 × 10^5^	0.1 × 10^5^	nd
Sample 1		0.6 × 10^5^	0.6 × 10^5^	—
Sample 2		1.0 × 10^5^	1.1 × 10^5^	—
Sample 3		1.2 × 10^5^	1.3 × 10^5^	—

Control	7	71.0 × 10^5^	4.0 × 10^5^	Nd
Sample 1		3.1 × 10^5^	3.0 × 10^5^	—
Sample 2		3.7 × 10^5^	3.8 × 10^5^	—
Sample 3		3.8 × 10^5^	3.6 × 10^5^	—

Control	15	3.9 × 10^5^	4.0 × 10^5^	—
Sample 1		4.8 × 10^5^	4.6 × 10^5^	—
Sample 2		5.0 × 10^5^	5.1 × 10^5^	—
Sample 3		5.3 × 10^5^	5.5 × 10^5^	—

Control	20	4.3 × 10^5^	4.4 × 10^5^	—
Sample 1		3.3 × 10^5^	3.2 × 10^5^	—
Sample 2		3.6 × 10^5^	3.5 × 10^5^	—
Sample 3		8.9 × 10^5^	9.0 × 10^5^	—

Abbreviation: nd, not detected.

### 3.9. Amino Acid Composition of Semidry Fermented and Smoked Sausages

Meat proteins are essential for maintaining protein homeostasis in the human body, compensating for continuous protein breakdown that occurs during metabolism. One of the key contributors to the taste and aroma profile of fermented meat products is the amino acid composition, as amino acids and their derivatives are formed during protein and peptide degradation in muscle tissue [60]. The results indicate that semidry fermented and smoked sausages treated with PAB exhibit a significantly higher content of essential amino acids compared to the control samples, with an overall increase of 24.2% in essential amino acid concentrations (*p* < 0.05). Table 8 presents the amino acid content and amino acid scoring values, demonstrating that valine, isoleucine, leucine, lysine, and threonine showed the highest relative increases. The accumulation of these amino acids is linked to the proteolytic activity of PAB, which influences both protein degradation and amino acid biosynthesis [4, 61, 62].

**Table 8:** Essential amino acid content and amino acid scoring values of semidry fermented and smoked sausage.

**Amino acids**	**Quantity (mg/100** g **of product)**	**Amino acid gradient (%)**
	**Control (± SD)**	**Fermented sausage (Sample 2) (± SD)**
Valine	836.45 ± 12.3	907.02 ± 14.1
Isoleucine	572.68 ± 10.8	701.10 ± 12.5
Leucine	1036.99 ± 15.6	1336.08 ± 18.2
Lysine	1026.00 ± 14.9	1383.85 ± 19.1
Methionine	367.65 ± 8.4	411.61 ± 9.7
Threonine	571.03 ± 9.9	705.71 ± 11.3
Tryptophan	169.03 ± 5.1	187.02 ± 5.6
Phenylalanine	497.99 ± 8.7	676.68 ± 10.2
Total	5077.82 ± 24.5	6309.08 ± 27.1

*Note:* Values are presented as means ± standard deviation (SD) from triplicate measurements (*p* < 0.05).

The predominant accumulation of glycine, glutamic acid, valine, phenylalanine, tyrosine, leucine, and isoleucine suggests that proteolytic enzymes, including endopeptidases and exopeptidases, are actively involved in the breakdown of proteins into free amino acids. Additionally, the biosynthetic activity of PAB further contributes to the increased free amino acid content, improving flavor development and textural properties [4, 63, 64].

The findings confirm that PAB significantly enhance the amino acid profile of semismoked sausages, particularly essential amino acids, which play a crucial role in protein quality and sensory attributes. The fermented samples exhibited a notable increase in essential amino acids, indicating that starter cultures promote protein breakdown while simultaneously improving the bioavailability of key amino acids. These results align with previous research, which demonstrated that starter microorganisms enhance free amino acid and fatty acid composition, contributing to improved microbiological and organoleptic properties in fermented sausages [4, 62].

Although the study demonstrated elevated levels of free amino acids and improved texture in samples treated with starter cultures, a direct correlation analysis between proteolysis and textural modifications was not conducted. Considering the known influence of proteolytic activity on meat texture, this relationship merits further investigation in future research.

## 4. Conclusion

The incorporation of *P. shermanii* and *L. acidophilus* as starter cultures in semidry fermented and smoked sausages effectively enhanced product quality, with 0.1% inoculation yielding the most favorable results. This concentration improved acidification and color formation and allowed for a significant reduction in NaNO₂ without compromising safety or appearance. Treated samples showed higher levels of essential amino acids, better sensory profiles, and improved technological properties such as moisture regulation and structural integrity. Microbial assessments confirmed stable safety throughout refrigerated storage. Future studies should explore the impact of different fermentation durations, alternative thermal treatments, and additional probiotic strains to further refine product performance.

## Conflicts of Interest

The authors declare no conflicts of interest.

## Funding

No funding was received for this manuscript.

## Data Availability

Data are available on request from the authors.
